# Objective structural clinical examination for evaluating learning efficacy of Cultural Competence Cultivation Programme for nurses

**DOI:** 10.1186/s12912-020-00500-3

**Published:** 2020-12-01

**Authors:** Yu-Hsia Lee, Shu-Chuan Lin, Pao-Yu Wang, Mei-Hsiang Lin

**Affiliations:** 1 Department of Nursing, Mackay Memorial Hospital, Taipei, Taiwan, Republic of China; 2 Department of Nursing, Mackay Junior College of Medicine, Nursing and Management, Taipei, Taiwan, Republic of China; 3 School of Nursing, National Taipei University of Nursing and Health Sciences, Taipei, Taiwan, Republic of China

**Keywords:** Cultural competence, Objective Structured Clinical Examination (OSCE), Nurses, Standardised patient

## Abstract

**Background:**

Culture serves as an adhesive to bind the lives of people. There are no objective, useful tools to assess cultural competence and practice. In this study, we evaluated whether the cultural competence of nurses was strengthened through the Cultural Competence Cultivation Programme.

**Methods:**

A quasi-experimental research design was used to evaluate nurses working at a medical centre in Taiwan. They were randomly allocated into an experimental group (*n* = 47), which received the Cultural Competence Cultivation Programme, or a control group (*n* = 50), which did not receive the educational programme. After the intervention, learning efficacy of the participants was assessed using an Objective Structured Clinical Examination (OSCE). The research data were statistically analysed on SPSS.

**Results:**

The average score of the experimental group was significantly higher in the ‘communication ability and skill’ category. Furthermore, OSCE scores and Standardised Patient Survey assessment and total scores were significantly and positively correlated.

**Conclusion:**

The findings of this study can serve as a reference for designing future clinical education programmes.

## Background

Culture acts as an adhesive to connect the lives of people. Therefore, its impact on health should not be overlooked [1]. Nurses must be able to identify cultural differences and apply communication skills to provide person-centred nursing care to patients from various ethnicities. Cultural skills are the ability to leverage tools and resources to develop communication skills, gather information about an individual’s cultural background and conduct a cultural assessment accordingly to fulfil needs arising from the patient’s background [2]. Hospitals with culturally sensitive nurses and culturally friendly care environments have lower health inequalities and disparities [3–5]. Cultural competence is fostered through learning; thus, a fundamental strategy for nurses to foster cultural competence is participation in educational programmes [6]. Numerous earlier studies have proposed several factors that affect the cultural competence of nurses, including their ethnic minority background, interaction frequency with culturally different people at workplace or daily life, education level, experience of caring for multiple ethnic groups [7, 8], cultural diversity training [9, 10], language skills [8, 11] and clinical experience in foreign countries [12]. Furthermore, the amount of work experience and employment status will exert a direct impact on self-efficacy for cultural competence [13]. Because cultivation of cultural competence is an interactive process involving various cultural scenarios, a Cultural Competence Cultivation Programme must be multistrategic [14]. Jeffreys developed an educational programme involving different teaching strategies, such as narration, literature review and discussion, film appreciation, and roleplaying, to help clinical nurses learn cross-cultural nursing concepts and assess their cultural competence [15]. Several studies have suggested considering course evaluation, performance analysis, and continuous course adjustment for designing an educational course that helps cultivate the cultural competence of nurses and for accumulating empirical data on cultural competence [4, 16].

Miller asserted that a bottom-up approach to determine professional competence follows the assessment of knowledge, know-how, show-how, and do [17]. The Objective Structured Clinical Examination (OSCE) is a show-how approach to assess the professional execution of learner skills [18]. In other words, it is a competence-oriented assessment of clinical performance. As a single, consolidated test, the OSCE can be used to objectively, fairly and safely assess student knowledge, skills and attitudes in a simulated environment, enabling learners to confidently prepare for future clinical encounters [16, 19]. It uses a reliable and valid categorical, structured checklist and standardised patients (SPs) to test learners on specific scenarios and standard operating procedures in controlled situations [16]. Assessment criteria for the OSCE are interpersonal communication, clinical problem-solving competence, health education, evaluation and decision-making abilities [15, 20, 21]. The OSCE is increasingly becoming the primary tool to assess clinical competence [20, 21]. Moreover, to value patient safety, the OSCE is not administered on actual patients. By adopting the objective and structured characteristics of SPs and using well-designed clinical scenarios, the OSCE is more capable of assessing learner clinical competence than earlier traditional written tests. The test also allows for reflection and self-learning. Therefore, the OSCE more effectively enhances learning efficacy of learners than traditional tests [22]. Miller asserted that the SP-based OSCE is the best teaching tool for assessing ‘proper practices (performance)’ [17]. The OSCE has been applied in various simulated learning scenarios, including operational and communication skills, in medical and nursing education [23]. Bani-Issa et al. [24] administered the OSCE to students of a physical assessment programme to evaluate their physical assessment competence, and the students expressed that the OSCE promoted in-depth learning and prepared them for the real world. Solà-Pola et al. [25] invited nursing students to participate in qualitative interviews. The research subjects experienced the OSCE by engaging in educational activities, and the researchers were able to obtain desired clinical care performance data.

Ledford et al. [26] developed an innovative teaching method based on adult learning theory and social cognitive theory. The researchers combined the OSCE with the religious affiliations and spiritual issues of the respondents. The results indicated that the OSCE helps medical professionals foster skills necessary to communicate with patients in challenging situations. Developers of multicultural education programmes can design different courses, dialogues, and scenarios by applying the OSCE concepts: (1) providing standardised and diversified patients; (2) citing medical histories, conducting physical examinations, and providing nursing care; (3) presenting cultural discrepancies and focal cultural learning points as open questions; and (4) referencing anatomical systems, nursing care items, and learning objectives. The verbal and nonverbal expressions of the patient’s culture, such as word use, intonation, movement, gesture, and facial expression, can be examined to elucidate the appropriateness of caregiver responses in different cultural contexts [27]. The purpose of this learning process is to enhance the learner’s cultural observation, cultural sensitivity, cultural care performance, and reflection ability, thereby ensuring the provision of culturally appropriate nursing care and preparing students mentally and professionally to care for multicultural patients [28]. In recent years, the focus of medical education has shifted toward the assessment of clinical performance. However, evaluating cultural competence through a written examination is extremely challenging because there are no objective and useful tools to assess cultural competence. Therefore, we used the OSCE to evaluate the learning efficacy of nurses after completing a Cultural Competence Cultivation Programme.

## Methods

This study evaluated whether the cultural competence of nurses was strengthened through the Cultural Competence Cultivation Programme.

### Design and participants

We adopted a quasi-experimental research design to examine nurses working at a medical centre in Taiwan. The nurses were randomly allocated into an experimental group (*n* = 47), which participated in the Cultural Competence Cultivation Programme, or a control group (*n* = 50), which did not participate in an educational programme. For inclusion, participants had to be licensed nurses who graduated from an approved nursing programme and have served as a clinical nurse for at least 1 year. Nurses diagnosed as having cancer or depression were excluded from this study. G*Power (version 3.1) was used to calculate the required sample size. Two statistical tests were performed using an analysis of variance (repeated measures, between factors) with parameters α = .05 and power = 0.8. Cohen’s rule of thumb predicted an effect size of 0.25 and a moderate autocorrelation value of 0.5 at 50% time interaction. The minimum sample size was 82 participants. This value was adjusted to 100 to allow for a 23% loss rate (*n* = 23). In total, 97 respondents completed the formal intervention and the 2-month post-intervention OSCE (47 respondents in the experimental group and 50 respondents in the control group). The loss rate was 3%.

The basic attributes included age, gender, educational attainment, nursing seniority, professional rank, department of work, experience of caring for foreigners, experience of attending cultural courses, experience of studying or living abroad and language proficiency.

### Measures

#### Cultural Competence Cultivation Programme

The Cultural Competence Cultivation Programme for nurses is based on social learning theory and focuses on cultural awareness, cultural knowledge, cultural willingness, cultural skills, and cultural circumstances [29, 30]. The programme consisted of four units. In Unit 1, the authors introduced information on the cultural competence of clinical nurses and explained the procedures of the cultural competence education intervention programme. In Unit 2, experts with ample experience interacting with new immigrant families from diverse cultural backgrounds were invited. In Unit 3, the participants watched a movie on the racial war between two countries to encourage them to reflect on their ideas about culture. In Unit 4, the authors used structured role play activities to enable the participants to experience inequality in designed scenarios.

A new unit was taught each week and each unit lasted for 3 h, for a total intervention time of 12 h. Cultural competence courses with diverse teaching strategies, such as discussion, film appreciation, and roleplaying, and continual adjusting can enhance the cultural competence of clinical nurses. Three experts in the cultivation of cultural competence were invited to review and provide feedback on the cultural aspects of the programme. The programme was also adjusted based on feedback provided by the participants. The programme was delivered by two lecturers with postgraduate degrees in nursing practice and trained in multicultural education.

#### OSCE

Studies have reported that an education and assessment tool that combines Standardised Patient Survey (SPS) and OSCE can effectively evaluate the clinical performance of learners [31]. The OSCE was designed based on Khattab and Rawlins’s [32] recommendations. The OSCE comprised the Multicultural Objective Structured Examination (MOSE) to assess nurses and the SPS to assess standardised patients. The OSCE measurement was conducted a week after the intervention was completed.

#### MOSE

The evaluation criteria of the nurses involve the following: (1) doctor–patient communication, problem assessment and problem-solving; and (2) nursing instruction for medications (communication ability and skill). The test comprises 10 items. The items are scored on a 3-point scoring system, in which 2 denotes ‘accomplished’, 1 denotes ‘partially accomplished’ and 0 denotes ‘unaccomplished’. The scores of the 10 items are summed. A high score represents a high cultural competence in clinical care. The Cronbach’s α coefficient of the test is .70.

#### SPS

The contents of this survey include empathy and verbal and nonverbal communication skills. A score is allocated based on the perceived interactions between the SP and the respondent. In particular, 2 is allocated when the ‘correct’ field in the right column is checked, 1 for ‘partially correct’, and 0 for ‘not performed’. The fields represent the respondents’ perceived performance. The survey comprises five items, with the total score ranging from 0 to 10. A high score represents a high nurse-to-patient communication performance and cultural competence. The Cronbach’s α coefficient of the survey is .62.

All SPs who participated in the OSCE completed a general SP programme and a performance and teacher training programme (8 h) and an OSCE rehearsal (3 h). Before the lesson, the researcher and three SPs discussed their roles. Before commencement, the examiner and the SPs discussed the script and rehearsed the scenario. The OSCE was administered 2 months after the completion of the education programme. The two groups of respondents were telephonically informed that they could take the test at the Clinical Competence Centre. The respondents entered the examination hall in order of registration, regardless of the group. The examiner and SPs could not identify the respondent groups, which ensured the research results remained unbiased.

The validity of the OSCE has been assessed by experts with over 10 years of medical and clinical education experience based on the content validity index (CVI). A four-point scoring system was adopted as the assessment standard. Expert opinions were consolidated and applied and referenced to adjust the research tools [33]. The item-CVI and scale-CVI coefficients were 1.00 for the MOSE and SPS.

### Data collection

The study was conducted from August 2017 to July 2018. The standard procedures of the OSCE were based on those recommended by Boursicot and Roberts [29].

#### Designing and editing lesson plans

Several meaningful real-world examples were used to design the programme scripts. Lessons were classified into the following segments: (1) student guidelines, involving patient background data, clear instructions, and test time; (2) examiner guidelines, involving case descriptions, patient summaries, health education tools and models, and scoring standards; (3) SP guidelines, involving basic SP information, script summaries, and dialogues; and (4) score sheet, involving the assessment items, content, and scoring standards.

#### Examiner consensus

A teaching video was developed and evaluated collaboratively by the examiners. The Cronbach’s α coefficient was 0.85, indicating a fair internal consistency.

#### OSCE operating procedures

The Clinical Competence Centre of the research hospital served as the OSCE administration centre. The examination was conducted in a simulated clinical environment that could be unidirectionally monitored and recorded. The script and notes were stuck to the door. The scenario involved a 23-year-old Vietnamese caregiver who has worked in Taiwan for 2 months. Her role was to provide care for a 75-year-old man with stroke experiencing a urinary infection. The patient is scheduled for discharge that day. Student guidelines included background information, test topics, and test time. The scene involves a nurse delivering post-discharge instructions to the Vietnamese caregiver regarding medications prescribed to the patient. During the examination, the examiner completed the MOSE based on the examiner guidelines. After the examination, the SPs completed the SPS based on their perceived subject performance.

### Data analysis

The data were processed and analysed using SPSS for Windows (version 22.0). Demographics were analysed using a descriptive statistics approach. The results are presented as percentages, mean values, and standard deviation values. Independent *t* test was used to compare intergroup differences. The correlations between the assessment tools in the OSCE were determined using a Spearman’s correlation analysis. A *p* value of <.05 was adopted as the measure of statistical significance.

### Ethical considerations

This study was approved by the McKay Memorial Hospital Institutional Review Board of the research hospital (Approval No. 17MMHIS096e). Before the study initiation, the participants were fully informed of the research objectives and data collection methods. They could withdraw from the study at any time for any reason. The data collected were archived anonymously. The study was initiation once the participants provided their consent and signed the consent form.

## Results

### Basic attributes of the research subjects

The average age of the participants was 36.49 (±10.14) years, whereas that of the experimental group participants was 35.98 (±9.99) years. Overall, the participants had an average of 14.78 (±10.31) years of nursing seniority. The experimental group participants had an average of 14.40 (±10.17) years of nursing seniority. Regarding educational attainment, 68% of the participants were university graduates. Regarding professional rank, 35.1% of the participants attained a rank of N4. Regarding the department of work, 36.1% of the participants served in internal medicine. However, there was no significant statistical difference between the two groups (*p* > .05) in any of the aspects, except for the department of work (Table 1).

**Table 1:** Basic attributes of participants

Variables		Experimental group (*n* = 47)		Control group (*n* = 50)		Homogenous test		Total (*n* = 100)	
		n	%	n	%	χ^2^	*p*	n	%
Gender	Female	47	100	50	100			97	100
Educational attainment	Diploma	12	25.5	16	32.0	.05	.97	28	28.9
	Bachelor’s degree	32	68.1	34	68.0			66	68.0
	Master’s degree	3	6.4	0	0			3	3.1
Department of work	Internal medicine	20	42.6	15	30.0	16.74	.005	35	36.1
	Surgery	9	19.1	0	0			9	9.3
	Obstetrics & Gynecology	5	10.6	10	20.0			15	15.5
	pediatric	5	10.6	5	10.0			10	10.3
	Intensive care unit	3	6.4	10	20.0			13	13.4
	other	5	10.6	10	20.0			15	15.5
Professional rank	N2	17	36.2	18	36.0	.05	.97	35	36.1
	N3	14	29.8	14	28.0			28	28.9
	N4	16	34.0	18	36.0			34	35.1
Language proficiency	no	36	76.6	41	82.0	.43	.51	77	79.4
	yes	11	23.4	9	18.0			20	20.6
Experience in caring for foreigners	no	3	6.4	4	8.0	.09	.75	7	7.2
	yes	44	93.6	46	92.0			90	92.8
Experience of attending cultural courses	no	45	95.7	47	94.0	.15	.69	92	94.8
	yes	2	4.3	3	6.0			5	5.2
Experience of studying or living abroad	no	43	91.5	49	98.0	2.10	.14	92	94.8
	yes	4	8.5	1	2.0			5	5.2
		Mean	SD	Mean	SD	*t*	*p*	Mean	SD
Age		35.98	9.99	37.00	10.37	−.50	.61	36.49	10.14
Nursing seniority (year)		14.40	10.17	15.16	10.54	−.36	.71	14.78	10.31

### OSCE intergroup effect analysis

The OSCE scores of the two groups were submitted to intergroup differential analysis. In the MOSE, the average scores of the experimental and control groups were 1.83 (SD = 0.14) and 1.78 (SD = 0.18), respectively. The average score of the experimental group was slightly higher than that of the control group, although the difference was nonsignificant (*p* = .08). The average scores for doctor–patient communication, problem assessment, and problem-solving were 1.91 (SD = 0.13) and 1.90 (SD = 0.13) for the experimental and control groups, respectively. However, the difference did not achieve statistical significance (*p* = .086). The average scores for nursing instructions on medication (communication ability and skill) were 1.72 (SD = 0.24) and 1.59 (SD = 0.30) for the experimental and control groups, respectively. The average score of the experimental group was slightly but significantly higher than that of the control group (*p* = .01). The average score of the experimental group for the “explaining the precautions for taking medication” item was also slightly higher than that of the control group, and the difference achieved significance (*p* < .001; Table 2).

**Table 2:** Intergroup differential analysis of the OSCE scores of the two groups

Variables	Experimental group		Control group		*t*	*p*
	Mean	SD	Mean	SD		
Doctor-patient communication, problem assessment, and problem-solving	1.91	.13	1.90	.13	.17	.86
•Shows respect for foreign caregivers (look and tone) and a sincere attitude	1.96	.20	1.96	.19	−.06	.95
•Shows empathy (listens attentively without interrupting the speaker)	1.91	.28	1.88	.32	.56	.57
•Uses language that the patient understands, speaks at a suitable speed, and uses appropriate body language	1.83	.38	1.88	.32	−.69	.48
•Solicits a response or validation (responds appropriately to the patient’s queries and verifies that the patient understands)	1.85	.36	1.84	.37	.15	.88
•Able to appease foreign caregivers	1.91	.35	1.90	.303	.22	.82
•Able to understand the foreign caregiver’s queries, identify the problem, and provide a valid explanation	2.00	.00	1.98	.141	.96	.33
Nursing instructions on medication (communication ability and skill)	1.72	.24	1.59	.30	2.47	.01
•Able to explain the precautions for taking medication	1.57	.50	1.16	.37	4.61	< .001
•Able to understand the foreign caregiver’s queries, identify the problem, and provide a valid explanation	1.72	.45	1.70	.46	.25	.80
•Able to confirm that the foreign caregiver understands administration procedures	1.85	.36	1.84	.37	.14	.88
•Able to employ guided or alternative methods of explanation (e.g., pictures, images, actions, talking speed)	1.77	.42	1.66	.519	1.09	.27
Total MOSE score	1.83	.14	1.78	.18	1.72	.08

*MOSE* Multicultural Objective Structured Examination, *OSCE* Objective Structured Clinical Examination

### Differential analysis of SPS scores and correlations between the various assessment tools of the OSCE

The intergroup chi-square test comparing the experimental and control groups did not demonstrate any significant statistical differences. Regarding the correlations between the various assessment tools of the OSCE score, the overall MOSE score and the SPS assessment score had a significant and positive correlation (*r* = .34, *p* < .01), the overall MOSE score and the overall SPS performance score had a significant and positive correlation (*r* = .36, *p* < .01), and the SPS assessment score and the SPS performance score had a significant and positive correlation (*r* = .95, *p* < .01; Table 3).

**Table 3:** Correlations between the various assessment tools of the OSCE

variables	1	2	3
1. OSCE scores	1		
2. SPS assessment score	.34^**^	1	
3. overall MOSE score	.36^**^	.95^**^	1

** *p* < .01

## Discussion

In this study, the OSCE was used to assess the effectiveness of a Cultural Competence Cultivation Programme for nurses. After participating in the Cultural Competence Cultivation Programme, there was a statistically significant difference in the MOSE score for ‘nursing instructions on medication (communication ability and skill)’, with the experimental group achieving a higher score than the control group. This finding is similar to the results of several previous studies. Alinie et al. [34] examined differences in the practical abilities of nurses after they had received situational simulation training. In their study, the experimental group received situational training and the control group received traditional classroom lessons and clinical training. The OSCE assessment for learning efficacy indicated that the experimental group significantly outperformed the control group. Moreover, studies have reported that the OSCE provides a controlled environment to train instructors in achieving a common teaching goal. Compared to conventional assessment methods, the OSCE is more capable of enhancing the learning efficacy of the learners because it provides intricately designed clinical situations that enable examiners to observe learner nursing performance on SPs and allow learners to reflect and learn from their experiences [22, 35]. The outcomes of the aforementioned studies coincide with the findings of this study, particularly those pertaining to the performance of ‘nursing instructions on medication’. However, our findings are different from those of Doyle et al., who designed a nursing education programme for care of patients with communication difficulties [36]. In their study, the experimental group received communication training and the control group did not receive any intervention. The OSCE was used both in pretests and posttests. The findings revealed an improvement in communication, self-efficacy, and OSCE performance of the experimental and control groups. However, the level of improvement between the two groups had no significant difference. Doyle et al. attributed their findings to both groups receiving the OSCE before the intervention and concluded that the OSCE effectively improved communication self-efficacy and OSCE performance.

The total MOSE score and the ‘doctor–patient communication, problem assessment, and problem-solving’ score of the experimental group were higher than those of the control group. However, the scores of the two groups failed to achieve significant statistical differences (*p* > .05). Most students expressed that the assessment method of OSCE was stressful. This method could have produced biases in respondent performance. The Cronbach’s α coefficient of the SPS was .62. Assessment of communication skills, single-station OSCE, and low item count may have influenced the internal consistency and reliability. A systematic review of the validity of the OSCE scores indicated that the covariates with significant Cronbach’s α were OSCE content (clinical scale higher than communication scale), the number of examiners (two higher than one), and scale type (checklist higher than Likert scale) [37]. During the OSCE, the respondents entered the examination hall in order of registration regardless of their group. The examiner and SPs could not identify the respondent groups, which ensured that the research results remained unbiased [38].

The examiners of this study collaboratively assessed an educational video. Cronbach’s α coefficient was used as an indicator of homogeneity. The results indicated a Cronbach’s α coefficient of 0.85, which was within the acceptable range. A coefficient value of <.7 signifies that the homogeneity of a some items is questionable, requiring modifications. A coefficient of >.9 signifies the excellent internal consistency or homogeneity of the items [39]. The results revealed that the total OSCE score was significantly and positively correlated with the SPS assessment and total scores. These findings are different from those in the study by Schwartzman et al. [40] involving pharmacy students whose communication skills were assessed using the OSCE. The evaluation was conducted by the invited examiners and SPs. Their findings indicated no significant correlation between examiner assessments and SP assessments. The lack of significant correlation could be attributed to the different roles of the SPs and examiners. SPs are the direct subject of communication in the examination, whereas examiners are third-party observers. Hence, SPs and examiners may perceive the learner’s communication skills differently. Jeffreys highlighted that educators must evaluate courses to determine their efficacy and continue to adjust them so as to increase experimental data on developing cultural competence [15]. In the course content designed for future research, cultural content can be enhanced to include different views of patients on health concepts and treatment methods to help participants learn effectively. Several limitations to the present study need to be addressed. First, a review of the OSCE items and examiner reliability and validity suggested that the test environment and line arrangement influence the reliability and validity of the OSCE. These factors were not addressed in this study, which is a limitation. In addition, the OSCE is resource-intensive. The high cost of conducting OSCEs has limited its application. We recommend that future research focus on the external validity of different OSCEs. Third, there was the potential for cross-contamination as the participants were recruited from a single hospital. Future research considers random allocation of the participants to different hospitals, which will help reduce cross-contamination. Fourth, as noted earlier, the outcomes were measured 1 week after the completion of the Cultural Competence Cultivation Programme because of time limitations. We hope that use of the OSCE will assist in assessing major changes, but longer time and repeated follow-up actions may lead to different findings, and future studies can consider this option. Finally, this study did not investigate the cultural background of the nurses, but it only tested the homogeneity of the basic attributes between the two groups. In the future, we should understand the cultural background of the nurses, conduct a homogeneity test of cultural competence, understand the influencing factors of cultural competence, and ensure that there is no difference between the groups.

## Conclusion

In this study, we designed an approach to determine the performance of education programmes. The proposed approach was employed to efficiently assess the cultural competence of clinical nurses. The findings of this study serve as a reference for health care providers in the process of designing cultural competence-related education programmes to improve clinical nursing care quality.

## Data Availability

The datasets used and analysed during the current study are available from the corresponding authors on reasonable request.

## Abbreviations

OSCE: Objective Structured Clinical Examination
SPS: Standardized patient survey
SPs: Standardized patients
MOSE: Multicultural Objective Structured Examination
CVI: Content validity index
SP: Standardized patient
